# Beyond the Kidney: A Systematic Review of Extrarenal Manifestations in *MAPKBP1*-Related Nephronophthisis

**DOI:** 10.3390/ijms27188370

**Published:** 2026-09-20

**Authors:** Enrico Ambrosini, Anita Luberto, Sabrina Busciglio, Maria Chiara Baroni, Antonietta Taiani, Ilenia Rita Cannizzaro, Ilaria Gandolfini, Umberto Maggiore, Davide Martorana, Vera Uliana, Valeria Barili, Antonio Percesepe

**Affiliations:** 1 Medical Genetics, University Hospital of Parma, 43126 Parma, Italy; eambrosini@ao.pr.it (E.A.);; 2 Department of Medicine and Surgery, University of Parma, 43126 Parma, Italy; 3 Nephrology Unit, University Hospital of Parma, 43126 Parma, Italy

**Keywords:** nephronophthisis, *MAPKBP1*, phenotype expansion, rare diseases

## Abstract

Nephronophthisis type 20 (NPHP20; MIM #617271) is a rare autosomal recessive disorder caused by biallelic pathogenic variants in *MAPKBP1*. Although traditionally considered a kidney-restricted disorder, recent findings suggest that *MAPKBP1*-related disease may be associated with a broader spectrum of extrarenal manifestations. However, the available evidence remains fragmented across gene-discovery studies, family reports, diagnostic sequencing cohorts, and functional investigations. This systematic review aimed to characterize the extrarenal manifestations of *MAPKBP1*-related disease and estimate their frequency. A secondary objective was to describe the spectrum of pathogenic or likely pathogenic *MAPKBP1* variants and integrate clinical findings with current mechanistic models. Studies were eligible if they reported at least one patient with biallelic *MAPKBP1* variants and provided clinical information relevant to the phenotype. PubMed, Embase, ClinVar, and Google Scholar were systematically searched from inception to August 2026. The methodological quality and risk of bias of included reports were assessed using the appropriate Joanna Briggs Institute (JBI) critical appraisal tools. All included reports were judged to be of high or moderate methodological quality. Only one paper was considered at high risk of bias, while the remaining articles or database entries had a moderate or low risk of bias. ClinVar entries were evaluated separately, with additional information obtained from the respective submitters when required. Clinical and molecular data were extracted and descriptively synthesized across included reports. The frequency of individual extrarenal manifestations was calculated based on the total number of identified patients while considering possible reporting biases. The search identified 154 records, including duplicates. After deduplication, 100 records underwent title and abstract screening and 32 full-text reports were assessed for eligibility. Nine articles met the inclusion criteria, comprising 16 patients; three additional patients were identified through ClinVar, yielding a total of 19 patients with *MAPKBP1*-related nephronophthisis included in the analysis. Most patients presented with at least one extrarenal manifestation, predominantly involving the skeletal system. Scoliosis was the most frequently reported extrarenal feature, occurring in eight of 19 genetically eligible patients. Other reported manifestations included short stature, patellar subluxation, long fingers or feet, craniofacial dysmorphisms, and cardiovascular abnormalities. The identified pathogenic or likely pathogenic *MAPKBP1* variants were predominantly stop-gain, splice-site, and frameshift variants. The evidence was limited by the rarity of *MAPKBP1*-related disease, the small number of reported patients, heterogeneity in clinical assessment and reporting, and the predominance of observational and case-based evidence. This systematic review supports an expanding extrarenal phenotypic spectrum of *MAPKBP1*-related nephronophthisis, with skeletal abnormalities (e.g., scoliosis) representing a prominent feature. Further systematic phenotyping and genotyping of affected individuals are warranted to formally suggest multidisciplinary surveillance, to clarify genotype–phenotype correlations and to investigate the mechanisms underlying extrarenal disease. Registration: The review was prospectively registered in PROSPERO (CRD420261429078).

## 1. Introduction

Nephronophthisis (NPHP) comprises a genetically heterogeneous group of autosomal recessive tubulointerstitial kidney disorders and represents an important inherited cause of kidney failure in children, adolescents, and young adults. The disease is characterized by an insidious loss of urinary concentrating ability, polyuria, polydipsia, anemia, growth impairment, and a progressive decline in kidney function. Histopathological findings commonly include tubular basement membrane abnormalities, tubular atrophy, interstitial fibrosis, and, particularly in advanced disease stages, corticomedullary cyst formation [1,2].

Although kidney involvement may occur in isolation, NPHP may also occur as part of a broader spectrum of nephronophthisis-related ciliopathies with variable extrarenal involvement [2]. Associated manifestations may include retinal degeneration, cerebellar or brainstem abnormalities, developmental delay, hepatic fibrosis, and skeletal defects. This phenotypic variability can complicate clinical recognition and genotype–phenotype interpretation [1,3].

Most NPH-associated proteins localize to the primary cilium, basal body, transition zone, centrosome, or related cellular compartments. These proteins participate in ciliary assembly, signal transduction, cytoskeletal organization, cell-cycle regulation, and the maintenance of tubular epithelial architecture. However, NPHP pathogenesis may also involve alterations in centrosomal organization, intracellular trafficking, and cell-cycle regulation, suggesting that different molecular defects can converge on a common tubulointerstitial phenotype [1].

### 1.1. Identification of MAPKBP1 as a Nephronophthisis-Associated Gene

*MAPKBP1*, also referred to as *JNKBP1*, emerged as a nephronophthisis-associated gene following the identification of biallelic loss-of-function variants in individuals with juvenile- or late-onset chronic tubulointerstitial kidney disease. In the original report by Macia et al., affected individuals from several unrelated families presented with a predominantly renal phenotype characterized by progressive chronic kidney disease, limited or absent proteinuria, tubulointerstitial fibrosis, and variable progression to kidney failure [4]. This condition was subsequently defined as “Nephronophthisis type 20” (NPHP20, MIM #617271).

The clinical presentation differed in some respects from the severe childhood-onset forms associated with several other NPHP genes. In particular, *MAPKBP1*-related disease appeared to be associated with relatively late recognition and, in some individuals, a slow progression of kidney dysfunction [4]. Subsequent reports identified additional affected families and expanded the spectrum of pathogenic *MAPKBP1* variants, including nonsense, frameshift, splice-site, and missense changes [5,6,7,8]. These observations also indicated that *MAPKBP1*-related nephronophthisis should be considered in adolescents and adults with unexplained chronic tubulointerstitial kidney disease, particularly when proteinuria is minimal and conventional imaging findings are nonspecific [5,6].

Initial functional studies generated particular interest because the MAPKBP1 protein could not be detected at the primary cilium, while patient-derived fibroblasts did not show an overt reduction in the proportion of ciliated cells [4]. On this basis, *MAPKBP1*-related nephronophthisis was initially proposed as a potentially cilia-independent form of NPHP [4]. This interpretation distinguished MAPKBP1 from many established NPHP proteins and raised the possibility that disruption of noncanonical signaling or centrosomal processes could produce an NPHP-like renal phenotype independently of a primary defect in ciliogenesis.

### 1.2. MAPKBP1 as a Scaffold Protein of the JNK Signaling Pathway

Before its association with kidney disease, *MAPKBP1* had been identified as a binding partner and putative scaffold protein of the c-Jun N-terminal kinase (JNK) signaling pathway [9]. The JNK pathway is one of the major branches of mitogen-activated protein kinase signaling and regulates cellular responses to stress, inflammation, DNA damage, cytoskeletal perturbation, proliferation, differentiation, and apoptosis. Scaffold proteins contribute to the spatial and temporal control of this pathway by coordinating interactions among kinases and their upstream or downstream partners.

MAPKBP1 is a large multidomain protein containing multiple WD40 repeats and a C-terminal domain involved in protein–protein interactions. These structural features are consistent with a scaffolding function and may enable MAPKBP1 to organize distinct signaling complexes within specific subcellular compartments [9,10]. MAPKBP1 has also been shown to interact with *WDR62*, another JNK-associated scaffold protein with important roles in centrosomal organization, spindle-pole integrity, and neurodevelopment [10]. Cohen-Katsenelson et al. identified a conserved dimerization domain shared by MAPKBP1 and WDR62 and demonstrated that scaffold-protein dimerization contributes to the recruitment and organization of JNK-pathway components [10].

Moreover, MAPKBP1 was identified as a scaffold protein that binds JNK isoforms and enhances JNK activation induced by MEKK1 and TGF-β-activated kinase 1 (TAK1) [9]. Subsequent work demonstrated that MAPKBP1 interacts with TAK1 and the upstream adaptors TRAF2 and TRAF6, enhances TAK1/TRAF-dependent NF-κB activation, and promotes TRAF2 polyubiquitination [9,11]. Independently of canonical SMAD2/3 signaling, activated TGF-β receptors can engage a non-canonical TRAF6–TAK1 pathway, leading to the activation of JNK and p38 MAPKs [12]. These findings place MAPKBP1 at a signaling intersection that is directly relevant to both TGF-β responses and the spatial organization of stress-activated MAPK signaling.

The first disease-focused functional analyses suggested that pathogenic *MAPKBP1* variants may disrupt interactions of the MAPKBP1 protein with JNK and WDR62, impair localization to mitotic spindle poles, and alter cellular responses to DNA damage [4]. These findings supported a mechanistic model in which defective JNK scaffolding, centrosomal recruitment, or cell-cycle control could compromise the long-term integrity of renal tubular epithelial cells. However, the precise relationship between these cellular abnormalities and the development of chronic tubulointerstitial fibrosis remained unresolved.

### 1.3. From a Cilia-Independent Model to a Cilia–JNK Signaling Defect

Later studies substantially refined the initial cilia-independent model of *MAPKBP1*-related nephronophthisis. Schönauer et al. identified additional disease-associated *MAPKBP1* variants and demonstrated that protein dimerization is required for efficient centriolar recruitment [6]. Their findings placed *MAPKBP1* more directly within the centrosomal and ciliary cellular environment and showed that selected pathogenic variants impair dimer formation, protein stability, or recruitment to centrioles [6]. These observations suggested that the absence of overt ciliogenesis defects does not necessarily exclude a functional relationship between MAPKBP1 and the primary cilium.

More recent structure–activity analyses have provided further evidence linking *MAPKBP1* loss to altered ciliary signaling rather than to a complete failure of cilium formation. Findeisen et al. integrated clinical information from previously reported and newly identified individuals with functional analyses performed in patient-derived fibroblasts, overexpression models, and *MAPKBP1*-depleted cells [8]. The study showed that MAPKBP1 loss affects ciliary length, basal body localization, and trafficking of phosphorylated JNK in relation to the primary cilium [8]. These data support a model in which *MAPKBP1* participates in a cilia-associated JNK signaling axis required for the maintenance of renal tubular cell homeostasis.

Accordingly, *MAPKBP1*-related disease may be better interpreted as a disorder of ciliary signaling or cilium–centrosome-associated regulation rather than as a strictly cilia-independent nephropathy. This distinction is biologically relevant because ciliary dysfunction can involve abnormal signaling dynamics despite apparently preserved cilium formation. It also broadens the mechanistic framework of NPHP by indicating that disturbances in stress-activated kinase signaling, cytoskeletal organization, and centrosomal protein recruitment may converge on the fibrotic degeneration of renal tubules [6,8].

### 1.4. Rationale: The Emerging Clinical Spectrum of NPHP20

The number of individuals reported with biallelic *MAPKBP1* variants remains limited, and the clinical spectrum of the disorder is consequently still being defined. Available evidence consistently supports a predominantly renal phenotype, usually involving chronic tubulointerstitial kidney disease with minimal proteinuria and variable progression to kidney failure [4,5,6,8]. The most recent systematic clinical assessment suggested that *MAPKBP1*-related nephronophthisis may follow a distinctive natural history characterized, in several patients, by exceptionally slow disease progression compared with other genetic forms of NPHP [8].

Nevertheless, the age at onset, severity of kidney dysfunction, imaging findings, and timing of kidney replacement therapy vary considerably between reported individuals [4,5,6,7,8]. Some patients are diagnosed during childhood or adolescence, whereas others remain undiagnosed until adulthood. This variability may reflect differences in variant type and residual protein function; however, the currently available number of cases is insufficient to establish robust genotype–phenotype correlations.

The extent of extrarenal involvement is even less defined. *MAPKBP1*-related nephronophthisis was initially described as a non-syndromic or kidney-restricted disorder, and ocular abnormalities typically associated with other renal ciliopathies have not emerged as a recurrent feature [4,13]. However, isolated findings reported in individual patients, including skeletal, neurological, developmental, or other systemic manifestations, raise the question of whether the phenotype is truly restricted to the kidney or whether subtle extrarenal features have been underrecognized [5,6,8].

Large sequencing studies of patients with suspected nephronophthisis or broader ciliopathy phenotypes have also identified *MAPKBP1* variants, further supporting the inclusion of *MAPKBP1* in diagnostic gene panels [3,7,14]. However, these cohort-based studies often provide limited phenotypic detail, and not all reported variants are supported by comparable levels of segregation or functional evidence.

A systematic re-evaluation of published cases is therefore required to distinguish recurrent disease-associated features from isolated observations and to assess whether the current evidence may support an expansion of the *MAPKBP1* phenotypic spectrum and a consequent update of follow-up strategies.

## 2. Materials and Methods

### 2.1. Reporting Guideline and Protocol

Before starting the study, the PROSPERO and ClinicalTrials.gov registers were consulted, to avoid duplicates of ongoing studies. The present systematic review was then registered on the PROSPERO platform on the 23rd of June 2026, with the code CRD420261429078. The review protocol was uploaded to PROSPERO on the same date and may be accessed through this link: https://www.crd.york.ac.uk/PROSPEROFILES/1141ab29df4abc094260c8f5bb9af767.pdf (accessed on 23 June 2026). The registration on PROSPERO preceded the screening process and data selection: some changes are officially recorded on PROSPERO; other minor modifications are explained in the following sections.

The Preferred Reporting Items for Systematic Reviews and Meta-Analyses (PRISMA) 2020 guidelines were followed [15]. The PRISMA checklists (both for Abstract and for the systematic review) can be found in the Supplementary Materials (Tables S1 and S2). The search was focused on the extrarenal manifestations of *MAPKBP1*-related diseases and their possible impact on patients’ health and clinical follow-up.

### 2.2. Search Strategy

A comprehensive literature search was initially conducted using the PubMed and Embase databases. For the initial literature search, the exact search syntax entered into the search field was “MAPKBP1” with no Boolean operators, date, language, or other search restrictions applied. The initial literature search was performed on the 30th of June 2026 and included all records available up to that date, sorted by “Publication date” in PubMed and “Publication year” in Embase.

Additional records were identified using Google Scholar, including preprints and “grey literature”. Records were “sorted by relevance”, applying the following criteria: “Any time”, “Any type” and “Include citations”. Google Scholar does not provide an exact number of results; thus the first 100 results ranked by relevance were screened, a commonly adopted approach that provides broad coverage while maintaining a practical and focused screening process, particularly for very rare syndromes.

To account for alternative gene and disease nomenclature, the literature search was subsequently expanded to include “JNKBP1” and “NPHP20”. A combined search using the following syntax was performed in PubMed, Embase, and Google Scholar:

“MAPKBP1” OR “JNKBP1” OR “NPHP20”

All potentially relevant records retrieved through the expanded search were screened according to the predefined eligibility criteria. The expanded search was performed on the 24th of August 2026 and was considered the actual search strategy for this review. This constitutes a difference from the original review protocol on PROSPERO but did not lead to the inclusion of new studies (as explained later).

The ClinVar database was also searched for variants in the MAPKBP1 gene [16], applying the filter for Germline Classification to include only “Likely Pathogenic” or “Pathogenic” (class 4 or 5 according to ACMG guidelines). To exclude complex rearrangements, the filter “Short variant (<50 bps)” under “Variation size” was applied. The search on ClinVar was performed on the 30th of June 2026. Because ClinVar is a clinical variant database rather than a bibliographic database, it was treated as an additional source of evidence rather than as a literature database. ClinVar was independently queried to identify pathogenic or likely pathogenic MAPKBP1 variants and to detect potentially affected patients not identified through the literature search.

### 2.3. Eligibility Criteria

For the purpose of the present review, records were considered eligible for screening if they contained molecular or clinical data concerning the role of *MAPKBP1* in human ciliopathies. No language restrictions were applied during the initial searches of PubMed, Embase, or Google Scholar, but language was subsequently assessed as part of the title/abstract screening process. In detail, the following exclusion criteria were used in the phase of title/abstract screening:

• the title or abstract clearly focuses on non-human models (e.g., animals, plants).
• the title or abstract is written in languages other than English.

Records passing the initial screening went to full-text assessment, after which only articles or abstracts reporting at least one patient with homozygous or compound heterozygous variants in the MAPKBP1 gene were included in the review. This criterion was also applied to ClinVar, screening only the new variants that were clearly reported in a homozygous or compound heterozygous state in an affected patient.

Studies were excluded if the patient had other genetic diagnoses explaining the symptoms or had a single *MAPKBP1* variant in a heterozygous state. Molecular studies not involving new patients underwent full-text assessment and were used for the composition of the Section 1, but were not included in the final selection.

### 2.4. Selection Process

All records were randomly divided among three reviewers, so that each entry was independently screened by at least two of them. Duplicate records were identified manually by cross-checking the bibliographic information of all retrieved records across databases. Records referring to the same publication, identified based on matching titles, authors, journal, publication year, DOI, and other bibliographic details, were considered duplicates and retained only once. Publications retrieved from PubMed, Embase and Google Scholar were manually compared and deduplicated. To avoid double-counting of individual cases, the variants reported in ClinVar were also cross-referenced with the patients described in the included case reports and case series. ClinVar entries that could be traced to a patient already reported in an included publication were not considered. Additionally, records identified through Google Scholar were manually screened to identify conference abstracts and other grey literature reporting cases that had subsequently been published in full-text in PubMed or Embase-indexed articles. These records were excluded to avoid double-counting patients and/or cases already represented in the included publications. After duplicate removal, titles and abstracts were evaluated according to the predefined eligibility criteria. Clinical and molecular reports that were considered potentially relevant for the study underwent full-text assessment for eligibility. Studies fulfilling the inclusion criteria were included in the study. In case of disagreement between two reviewers, an opinion was sought from the third reviewer, in order to reach a majority decision.

### 2.5. Data Collection Process and Data Items

The complete report of each selected study was reviewed, and the following data were extracted: first author, year of publication, study design (experimental or clinical study), and main findings. Data from the reports including clinical and molecular information on new patients were collected in a spreadsheet containing the following data: case report/case series, sex, age at follow-up, type of genetic testing, genetic variants detected, detailed extrarenal manifestations. Every clinical feature was reported according to the nomenclature of the Human Phenotype Ontology (HPO). Similar terms were reported using the closest common tier (e.g., scoliosis and kyphoscoliosis). In the case of unspecific data items, the most generic HPO terms were applied (e.g., Abnormality of the face, Abnormal venous morphology...etc.).

### 2.6. Effect Measures, Synthesis and Statistical Analysis

Effect measures were extracted as originally reported in the included studies. Findings were synthesized descriptively and reported in tabular and graphical formats (see the Section 3). Descriptive statistics analysis was applied where feasible, namely chi-squared tests. Data were reported mostly as simple frequencies, since the expected small number of patients and the potential heterogeneity of data did not allow complex statistical analyses.

### 2.7. Quality Assessment and Risk of Bias

The quality and risk of bias assessments were performed only for the selected clinical studies. For this purpose, the Joanna Briggs Institute (JBI) Critical Appraisal Tools for Case Reports and Case Series were used [17]. When preparing the individual survey papers using the templates available on the JBI website (https://jbi.global/critical-appraisal-tools, accessed on 23 June 2026), three main possible sources of bias were identified:

• The inability to determine whether a clinical manifestation was absent in a patient, or simply not reported or not investigated using a specific diagnostic exam. This limitation was taken into account when calculating the frequencies.
• Application of a targeted panel instead of exome sequencing. The genetic testing strategy was defined for each report to assess whether other possible genetic diagnoses were excluded in relation to extrarenal manifestations.
• Conflicting nomenclature and classification of genetic variants. All variants were converted to the most up-to-date nomenclature using the transcript NM_014994.3 (MANE Select) according to GRCh38. They were revised by two independent reviewers applying the ACMG criteria, to assess bias related to patients with uncertain or likely benign variants.

These items were integrated alongside the original JBI criteria and were considered in the assessment of methodological quality and risk of bias. This adapted approach was employed to account for study-specific methodological features that were not fully captured by the original tools, while maintaining the overall structure and principles of the JBI critical appraisal framework. The tool for Case Reports was used for studies reporting only one family or reporting a wide heterogeneous cohort from which only one patient was extracted. The tool for Case Series was used for studies reporting two or more patients from different families. The assessment was performed independently by three reviewers, resolving disagreements through majority decision. The results were then summarized through tabulation.

### 2.8. Inclusion of New Patient Data

Additional clinical data was requested for patients reported in ClinVar: one of these patients was referred to our medical facilities, so the new data were added to those already described in the ClinVar entry. In compliance with the local ethical guidelines and the Declaration of Helsinki, the new patients included in this study provided informed consent for genetic analysis and results publication. Ethical review and approval were waived for this study according to the local policy; informed consent is considered sufficient for reports of an observational nature concerning a limited number of patients.

## 3. Results

### 3.1. Study Selection

**Figure 1:**
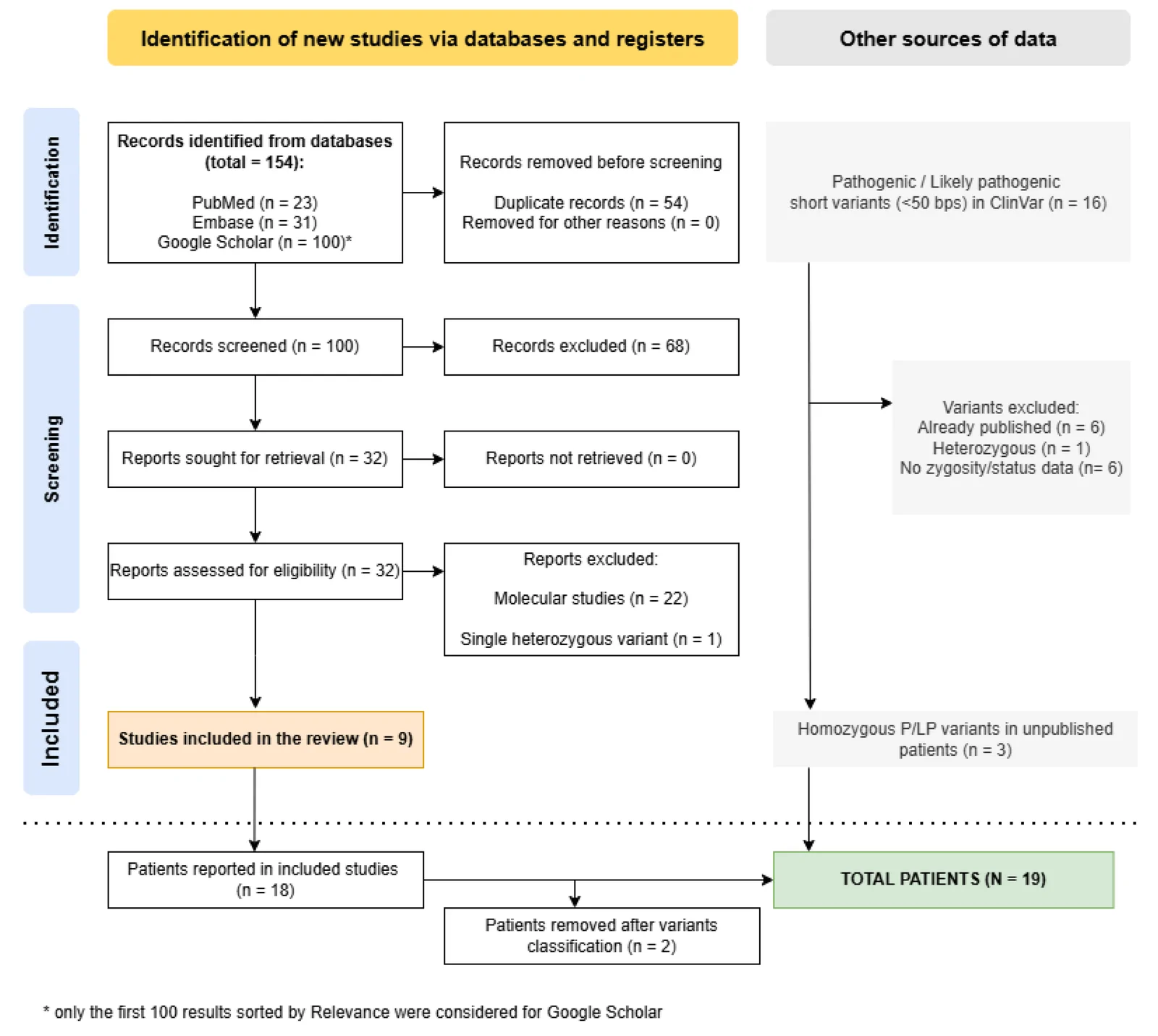
PRISMA flowchart illustrating the study selection process, created with the specific Shiny app [18] and then modified to include ClinVar and to specify the number of patients included. The dotted line separates the standard PRISMA flowchart from the outcome in terms of number of patients (a single study can report multiple patients). A total of 21 patients were identified, but 2 were removed for reasons explained in the main text, leading to a total of 19 reported patients.

The literature search identified a total of 154 records through electronic database searching. After removal of 54 duplicate records, 100 reports underwent title and abstract screening. Following this initial screening, 68 records were excluded because they did not meet the predefined eligibility criteria. A total of 32 full-text articles were sought for retrieval; however, 22 of them were classified as “experimental studies” and did not contain new patients. These studies were used to support the introduction and were cited accordingly but were not included in the final study selection. Another report was excluded since the reported patient carried only one heterozygous variant. The remaining nine reports were therefore included in the review. Three additional patients were identified through ClinVar in association with homozygous variants. The study selection process is illustrated in the PRISMA 2020 flow diagram (Figure 1).

### 3.2. Results of Quality and Risk of Bias Assessment

The nine selected articles and the three database entries reported a total of 21 patients from 17 different families. Two articles were actual case series with two or more patients from different families.

The appropriate JBI tools were used accordingly, and the results are summarized in the Supplementary Materials (Supplementary Tables S3 and S4). The majority (eight out of 12) of articles or database entries were of high or moderate quality (score > 5/8 for Case Reports and score > 6/10 for Case Series). Low scores was obtained for ClinVar entries (where JBI tools are not completely applicable) and for one preprint found through Google Scholar, mostly because of incomplete clinical data. The reviewers contacted the clinician for an Italian patient with incomplete data, while in the other cases additional data was sought in the grey literature (e.g., conference abstracts), with scarce results.

Even when clinical data were complete, the manifestations were often not listed as a timeline (only two cases were marked “Yes” for Question 2 in the Case Reports) or were only briefly summarized in the context of large cohort studies.

The risk of bias was assessed through the same tools, counting the questions answered with “No” or “Unclear”. Only one paper was considered at high risk of bias, while 7 articles or database entries had a moderate risk.

After the assessment, all authors re-evaluated the papers with “high” or “moderate” risk of bias and unanimously decided to exclude two patients, mostly because their *MAPKBP1* variants had been re-classified as variants of uncertain significance (VUS) or likely benign (see also the Genotype description section). One was a 2.5-year-old patient with pulmonary hypoplasia, enlarged echogenic kidneys with poor corticomedullary differentiation, and septal defects [14]. The other, reported in the preprint with low scores, was a 55-year-old Turkish man with focal segmental glomerulosclerosis and no reported extrarenal features [19]. While other types of bias could be addressed with proper adjustment, the inclusion of these two patients with uncertain diagnoses would have altered frequencies in a small cohort and/or led to incorrect interpretations.

### 3.3. Clinical Characterization of Included Patients

Since two patients (E01 and E02) were excluded, a total of 19 patients were officially included in the study. The mean age at last follow-up was 24.9 years, with a median age of 22 years; the youngest patient was 12 years old, but a patient was reported in ClinVar in the range of 0–9 years; the oldest was 47 years old. Males (n = 8) and females (n = 9) were similarly represented, while sex was not specified for two patients.

While renal involvement constituted the predominant phenotype in almost all patients, ranging from multiple cysts to chronic kidney disease, in 14 individuals (73.7%) at least one extrarenal clinical manifestation was reported. These features are summarized in Table 1.

**Figure 2:**
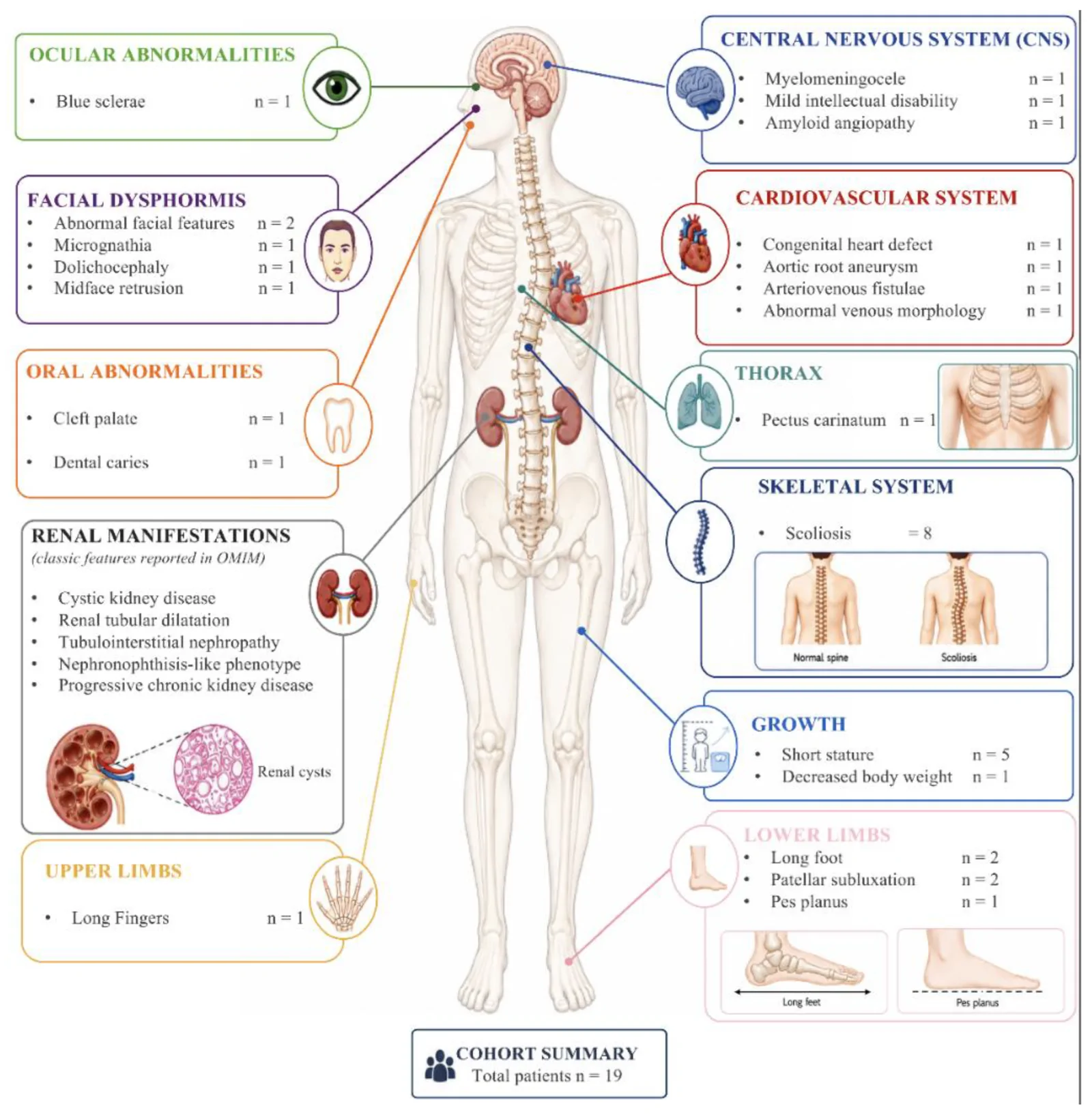
Schematic representation of the extrarenal phenotypic spectrum reported in individuals with *MAPKBP1*-related nephronophthisis. Clinical manifestations are grouped according to the main affected organ systems and anatomical regions, including ocular, craniofacial, oral, central nervous system, cardiovascular, thoracic, skeletal, growth-related, and upper- and lower-limb abnormalities. The number of patients in whom each feature was reported is indicated next to the corresponding manifestation.

During bias assessment, the answer to Q5 (“Was specific assessment for extrarenal features performed?”) was “Yes” for all included cases, since all contained direct or indirect references to an external clinical evaluation performed on the patient. Scoliosis was the most frequently reported extrarenal manifestation, reported in eight patients. It was not possible to define how many patients underwent X-ray or other specific imaging studies. Other recurrent features were short stature or growth delay (five patients, 26.3%), long fingers and/or foot (3/19, 15.7%), subluxation of the patella (2/19, 10.5%), and unspecified facial dysmorphisms. These findings are better summarized in Figure 2.

The skeletal and cardiovascular systems were the most frequently involved, with the overall phenotype resembling a connective tissue disease in some patients. Patient P06 [6] presented with severe scoliosis requiring surgical stabilization with Harrington rods. The habitus was described as marfanoid, and a kyphoscoliotic Ehlers–Danlos syndrome was suspected, but no additional potentially causative variants were identified through exome sequencing. A second patient from the same article, P07, had a vascular anomaly of the inferior vena cava, with partial malrotation to the left, merging into the left renal vein. Moreover, an additional patient reported in the ClinVar database [16] presented with scoliosis, short stature, patellar subluxation, pectus carinatum, midface retrusion, joint laxity and other features more commonly associated with Ehlers–Danlos syndromes.

The clinical and molecular data of another patient (P12) were recently submitted to ClinVar [16] by our Medical Genetics Unit. This was a 47-year-old man with nephronophthisis and consequent chronic kidney disease, which led to hemodialysis at the age of 36 years and renal transplantation at the age of 37 years. Ultrasound examination also revealed the presence of hepatic steatosis with small cysts. He required a full brace for severe dorsolumbar scoliosis and was subsequently diagnosed with osteoporosis (vertebral T-score −2.7, Z-score −2.4) associated with vertebral collapse. Cardiovascular evaluation revealed hypertrophy of the interventricular septum (15 mm), a dilation of the aortic root (44 mm), and fibrosis of the mitral valve. Additional vascular abnormalities were identified, particularly in the upper limbs, including a left humeral pseudoaneurysm, tortuosity of the forearm vasculature with an arteriovenous fistula, and a small aneurysm of the palmar tract of the ulnar artery. Considering the presence of mild dysmorphisms, including micrognathia, long fingers of the feet, and pes planus, a secondary diagnostic hypothesis was considered, leading to the investigation of collagenopathies and other connective tissue disorders. No other suggestive causative variants were found, apart from the biallelic pathogenic splice-site variant in *MAPKBP1*.

Regarding ocular manifestations, the only patient with retinitis pigmentosa also carried a pathogenic variant in *PDE6A*, which accounted for the ocular phenotype. Only one patient was diagnosed with mild intellectual disability [4].

### 3.4. Testing Strategies and Genotype Description

Whole exome sequencing (WES) or clinical exome was performed in 11 patients (52.3%). In three additional patients, a targeted panel focused on genes associated with renal diseases was used. Direct sequencing of known variants was performed in four individuals in the context of segregation analysis, while for the remaining three patients there were no data regarding the testing strategy.

Four patients had extrarenal symptoms but did not undergo exome sequencing to exclude a second diagnosis. No statistically significant difference in the frequency of reported extrarenal manifestations was observed between patients analyzed by exome sequencing and those analyzed using other methods (*p* = 0.62). However, given the small sample size, this finding should be interpreted cautiously and does not provide evidence of equivalence between the two groups.

Altogether, the 19 patients carried a total of 17 variants in the MAPKBP1 gene. Two other variants were described in a homozygous state in the two excluded patients and were classified as VUS or likely benign variants. In the ClinVar database, six other pathogenic or likely pathogenic variants are reported, but the zygosity and the phenotype were not described, except for one variant that was incidentally found in a heterozygous state in the context of expanded carrier screening. These 25 variants are listed in Table 2.

The allele frequencies of all pathogenic and likely pathogenic variants were checked in gnomAD v4 [21] and were summed in order to estimate the carrier frequency in the general population. The number of homozygotes was equal to zero for each considered variant. Based on the aggregate gnomAD allele frequency of established pathogenic/likely pathogenic variants (1.18 × 10^−5^), the estimated carrier frequency under Hardy–Weinberg equilibrium is approximately 2.36 × 10^−5^, corresponding to one in 42,400 individuals. This number is likely underestimated but is compatible with an ultrarare autosomal recessive condition.

Almost all variants were nonsense, frameshift or located in a splice-site. Only three missense variants were included, since they were reported in published patients. One of these, the p.Arg538Gln, was classified by both reviewers as likely pathogenic, applying PM2, PP5 and PS3 criteria. Indeed, well-established functional studies demonstrated a decrease in microtubule association, an increase in cytosolic abundance and a reduction in the green fluorescent protein intensity at the basal body of primary cilia relative to the cytosol, similar to the findings observed for truncating variants [8]. The patient (P03) carrying this variant in the homozygous state was reported to have extrarenal features and appeared to have a milder phenotype, with no progression to end-stage renal disease by the age of 23 years. Together with the functional studies reported by Findeisen et al., this observation may suggest a milder effect of missense changes; however, the number of available cases is too small to define a clear genotype–phenotype correlation.

The same functional experiments did not equally support a pathogenic role for another missense variant, the p.Arg1102Cys, which was also considered too frequent in gnomAD [21] and was therefore classified as likely benign (BS1, BP4). Finally, the p.Pro879Ser variant has not yet been investigated in functional studies, was predicted to be benign by multiple computational prediction tools, and was classified as variant of uncertain significance (PM2, BP4). Interestingly, the patient carrying this biallelic missense variant also did not show extrarenal features and would have been the oldest individual of the cohort (55 years). The two patients carrying these variants have been excluded from the study, as explained before.

## 4. Discussion

Despite growing interest in *MAPKBP1*-associated kidney disease, the available evidence remains fragmented across gene-discovery studies, individual family reports, diagnostic sequencing cohorts, and functional investigations. Consequently, several clinically and biologically relevant questions remain unresolved, including the typical age at disease onset, the frequency and nature of extrarenal manifestations, the pathogenic relevance of different variant classes, and the extent to which the emerging molecular data can explain clinical variability.

The evolving interpretation of the function of *MAPKBP1* also warrants a comprehensive reassessment. The disease was initially proposed as a cilia-independent form of nephronophthisis, whereas subsequent findings have implicated centriolar recruitment, protein dimerization, ciliary architecture, and cilia-associated JNK signaling [4,6,8]. Integrating these apparently divergent observations is necessary to better define the role of *MAPKBP1* within the molecular landscape of nephronophthisis and renal ciliopathies.

In the clinical dataset assembled for this review, scoliosis was reported in eight of 19 individuals with biallelic *MAPKBP1* variants, whereas Findeisen et al. had previously documented scoliosis in five of 16 published patients. Although potential ascertainment bias, non-standardized spinal assessment, age heterogeneity, and chronic kidney disease–mineral and bone disorder should be considered, the recurrence of scoliosis across unrelated families raises the possibility of a genuine phenotype expansion of the *MAPKBP1*-associated phenotypic spectrum. It was not possible to define how many patients underwent X-ray or other specific imaging studies, but all 19 patients underwent at least an external clinical evaluation: even as a cautious statement, it appears that scoliosis is frequent in patients with biallelic *MAPKBP1* pathogenic variants. In the *MAPKBP1* mutated cohort, the frequency of this manifestation (in eight out of 19 genetically eligible patients) can be estimated at approximately 40% (42.1%; exact 95% confidence interval: 20.3–66.5%). The repeated observation of scoliosis in multiple unrelated families and across different variant classes is unlikely to be fully explained by the expected background prevalence. Nevertheless, these estimates should not be interpreted as proof of causality. The *MAPKBP1* cohort is small and clinically heterogeneous, includes both pediatric and adult individuals, and absent manifestations may have been unreported rather than specifically excluded, while chronic kidney disease–mineral and bone disorder, secondary hyperparathyroidism, growth impairment, and reduced physical activity may act as confounders or modifiers. Accordingly, scoliosis is best considered a candidate for recurrent extrarenal manifestation that warrants prospective orthopedic assessment for proper clinical management of patients.

A possible cilia–TGF-β–JNK mechanism may be linked to the *MAPKBP1* deficiency-related scoliosis, even though the relevance to skeletal phenotype remains hypothetical. Originally, MAPKBP1 was identified as a scaffold protein that binds JNK isoforms and enhances JNK activation induced by MEKK1 and TGF-β-activated kinase 1 (TAK1) with a direct interaction with the upstream adaptors TRAF2 and TRAF6. These findings may position *MAPKBP1* at a signaling intersection that is directly relevant to both TGF-β responses and stress-activated MAPK compartmentalization, despite the ability of TGF-β to independently activate JNK and p38 through the TRAF6–TAK1 pathway. The only disease-relevant cellular data currently available come from Findeisen et al., demonstrating that MAPKBP1 is localized at the ciliary basal body in control cells and that the protein loss in patient-derived and cellular models is also associated with shortened primary cilia. JNK activation behaved as a molecular switch controlling MAPKBP1 association with microtubules, centrosomes, and basal bodies. Importantly, short-term TGF-β1 exposure reproduced *MAPKBP1* dissociation from the basal body and caused rapid ciliary shortening, whereas *MAPKBP1* loss altered the intracellular trafficking of phosphorylated JNK. On this basis, the most defensible interpretation is not simply excessive or deficient total JNK activity, but rather a loss of the normal spatial and temporal organization of JNK signaling at the cilium–centrosome interface [8]. These observations, however, were made in ciliated cells but not in bone-lineage-derived cells. Thereby, the extension of this mechanism to the skeletal phenotype relies on evidence from other unrelated experimental models, where primary cilia in osteoblasts and osteocytes are required for skeletal development and bone formation. The role of JNK in bone is strongly context dependent. JNK activity is required for ATF4 induction and late-stage osteoblast differentiation, and genetic studies support an important role for JNK1 in osteoblast function in vivo [22,23]. Conversely, JNK1 can negatively regulate BMP2-induced osteoblast differentiation through inhibitory phosphorylation of RUNX2 at Ser104 [24]. Separately, TAK1-dependent activation of p38 promotes RUNX2 transcriptional activity and is required for normal osteoblast differentiation, skeletogenesis, and bone homeostasis [25]. Critically, none of these studies examined the role of MAPKBP1 itself in bone-lineage cells. We therefore propose, as a testable hypothesis rather than a demonstrated mechanism, that MAPKBP1 deficiency may disturb the balance, duration, or subcellular localization of several TAK1-dependent outputs during osteoblast/chondrocyte differentiation and vertebral growth, resulting in defects in matrix deposition, cell polarity, or mechano-adaptation, possibly contributing to asymmetric loading and the development or progression of scoliosis.

Systematic orthopedic phenotyping and functional studies in *MAPKBP1*-deficient bone-lineage cells are required to better correlate osteo-phenotypes with a definitive molecular mechanism.

Short stature or growth delay was also a recurrent feature, affecting five patients. However, the specific height percentiles used to define short stature were not consistently reported. By assuming the standard inferior threshold of height below the third percentile, the observed frequency of 26.3% would be higher than the expected prevalence in the general population. Nevertheless, the same limitations considered for scoliosis may be applied here.

Several additional features commonly observed in connective tissue disorders, including blue sclerae, carious teeth, joint laxity, patellar subluxation, and long fingers, were also reported in *MAPKBP1*-related patients. The application of WES excluded a secondary genetic diagnosis in most of these individuals, but some conditions such as hypermobile Ehlers–Danlos syndrome, for which no definitive molecular diagnosis is currently available, cannot be excluded.

Regarding genotype–phenotype correlations, the common differences between truncating and non-truncating variants may also apply to *MAPKBP1*, with a less severe phenotype associated with missense variants. However, the available data are too limited to fully support this conclusion, so this observation has to be considered only “hypothesis-generating”. Further cases and functional studies are needed.

## 5. Conclusions

*MAPKBP1*-related nephronophthisis is primarily characterized by slowly progressive tubulointerstitial kidney disease, but the recurrence of bone-related phenotypes in reported individuals supports the possibility of an expanded phenotypic spectrum. The available molecular evidence suggests that the loss of *MAPKBP1* may disrupt ciliary localization and length, and the spatial control of JNK signaling in non-skeletal cellular models. Whether these abnormalities contribute directly to scoliosis or skeletal manifestations remains unknown. Further systematic phenotyping and comprehensive genotyping of affected individuals are warranted to delineate the full clinical spectrum and natural history of the disease, identify potentially underrecognized extrarenal manifestations, and provide a stronger basis for recommending multidisciplinary surveillance and management. Such efforts may also help refine genotype–phenotype correlations and shed light on the molecular mechanisms contributing to extrarenal involvement.

## Figures and Tables

**Table 1:** Extrarenal features of patients with biallelic variants in the MAPKBP1 gene.

Patient	Age	Sex	Test	Variants	Skeletal Features	Craniofacial Dysmorphisms	Cardiovascular Features	Other	Ref
P01-I	25	F	Exome	p.Arg198* p.Arg1459*	Patellar subluxation; Long fingers; Long foot	-	-	-	[4]
P01-II	27	M	Sanger	p.Arg198* p.Arg1459*	Short stature	-	Congenital heart defect	Myelomeningocele; (PDE6A-related retinitis)	[4]
P02	15	M	Exome	p.Arg434* p.Arg434*	-	Abnormality of the face	-	-	[4]
P03	23	M	Exome	p.Arg538Gln p.Arg538Gln	-	-	-	-	[4]
P04	15	F	Exome	c.2426-1G>A c.2426-1G>A	Short stature	-	-	-	[4]
P05-I	39	F	Exome	p.Gln937* p.Gln937*	Scoliosis	-	-	-	[4]
P05-II	38	M	Sanger	p.Gln937* p.Gln937*	Scoliosis	-	Amyloid angiopathy	Cholesteatoma; Vesicoureteral reflux	[4]
P05-III	21	F	Sanger	p.Gln937* p.Gln937*	Scoliosis; Short stature	Cleft palate	-	Mild intellectual disability	[4]
P06	37	F	Exome	c.4299+5G>C c.4299+5G>C	Scoliosis; Long fingers	Abnormality of the face		-	[6]
P07	14	F	Panel	c.3315-2A>G c.3315-2A>G	Short stature	-	Abnormal venous morphology	-	[6]
P08-I	31	M	Exome	p.Arg312* p.Arg312*	-	-	-	-	[5]
P08-II	12	M	Sanger	p.Arg312* p.Arg312*	-	-	-	-	[5]
P09	12	F	Panel	c.3314+1G>A p.Gln868*	-	-	-	(Hypokalemia)	[3]
P10	20	M	Panel	p.Gly752fs p.Gly752fs	-	-	-	-	[7]
P11	20	F	Exome	p.Ser826fs p.Ser826fs	Scoliosis	-	-	Decreased body weight; Cholelithiasis	[8]
P12	47	M	Exome	c.270-2A>G c.270-2A>G	Scoliosis; Long fingers; Long foot; Pes planus	Micrognathia	Aortic root aneurysm; Arterio- venous fistula	-	[16] SCV007606 611.1
P13	0–9	na	na	c.2885+2C>T c.2885+2C>T	Scoliosis	-	-	-	[16] SCV006333 410.1
P14	na	na	na	p.Ser424fs p.Ser424fs	Scoliosis; Short stature; Patellar subluxation; Pectus carinatum;	Dolichocephaly; Midface retrusion	-	Blue sclerae; Carious teeth; Joint laxity	[16] SCV002318 839.1
P15	47	F	na	p.Val1193fs p.Val1193fs	-	-	-	Polyarthritis	[20]
Excluded patients with variants reclassified as class 3 or class 2 according to ACMG criteria									
E01	3	na	Exome	p.Arg1102Cys p.Arg1102Cys	-	-	Congenital heart defect	(Pulmonary hypoplasia)	[14]
E02	55	na	Exome	p.Pro879Ser p.Pro879Ser	-	-	-	-	[19]

**Table 2:** List of *MAPKBP1* variants reported in affected patients, with their updated classification, or listed in ClinVar [16] as pathogenic or likely pathogenic. Variant nomenclature and population frequency refer to the transcript NM_014994.3 (MANE Select) according to GRCh38 genome assembly.

N	Variant (c.)	Protein (p.)	Type	ACMG Class	Allele Count	Allele Number	Allele Frequency
1	c.592C>T	p.Arg198*	Stop-gain	5	1	1,613,552	6.20 × 10^−7^
2	c.1270_1271del	p.Ser424Leufs*16	Frameshift	5	/	/	/
3	c.934C>T	p.Arg312*	Stop-gain	4	1	1,612,238	6.20 × 10^−7^
4	c.1300C>T	p.Arg434*	Stop-gain	5	2	1,614,208	1.24 × 10^−6^
5	c.1613G>A	p.Arg538Gln	Missense	4	2	1,611,462	1.24 × 10^−6^
6	c.1904+1G>A	p.?	Splicing	5	1	1,612,330	6.20 × 10^−7^
7	c.2092+1G>A	p.?	Splicing	5	/	/	/
8	c.2156+1G>A	p.?	Splicing	4	/	/	/
9	c.2227C>T	p.Gln743*	Stop-gain	5	1	1,614,226	6.19 × 10^−7^
10	c.2253del	p.Gly752Aspfs*74	Frameshift	4	/	/	/
11	c.2426-1G>A	p.?	Splicing	5	/	/	/
12	c.2476_2480del	p.Ser826Glyfs*34	Frameshift	4	/	/	/
13	c.2602C>T	p.Gln868*	Stop-gain	4	/	/	/
14	c.270-2A>G	p.?	Splicing	4	/	/	/
15	c.2809C>T	p.Gln937*	Stop-gain	5	/	/	/
16	c.2885+2C>T	p.?	Splicing	5	/	/	/
17	c.3314+1G>A	p.?	Splicing	4	/	/	/
18	c.3315-2A>G	p.?	Splicing	4	/	/	/
19	c.3428del	p.Pro1143Argfs*186	Frameshift	5	/	/	/
20	c.3578_3579del	p.Val1193Alafs*10	Frameshift	4	/	/	/
21	c.4299+1G>A	p.?	Splicing	4	/	/	/
22	c.4299+5G>C	p.?	Splicing	4	1	1,588,072	6.30 × 10^−7^
23	c.4375C>T	p.Arg1459*	Stop-gain	4	10	1,612,074	6.20319 × 10^−6^
** **	**Reclassified variants (not considered for frequency counts)**						
1	c.2635C>T	p.Pro879Ser	Missense	3	1	1,614,156	6.19519 × 10^−7^
2	c.3304C>T	p.Arg1102Cys	Missense	2	135	1,613,918	8.36458 × 10^−5^

## Data Availability

All relevant data are available from the corresponding author upon request. The new pathogenic variants have been reported in ClinVar public database. The review protocol is available on the PROSPERO registry.

## Supplementary Materials

The following supporting information can be downloaded at https://www.mdpi.com/article/10.3390/ijms27188370/s1. Reference [26] is cite in Supplementary Materials.
