# Supplementary material for: Beyond the Kidney: A Systematic Review of Extrarenal Manifestations in MAPKBP1-Related Nephronophthisis
Source: Int J Mol Sci. 2026 Sep 20;27(18):8370. doi: 10.3390/ijms27188370 (PMC13607008; doi:10.3390/ijms27188370)
Supplement: Supplementary file 1 [file ijms-27-08370-s001.zip › ijms-4519740-supplementary.pdf]

Supplementary Table S1. PRISMA 2020 checklist for Abstracts.

Section and Topic	Item #	Checklist item	Reported
**TITLE**			
Title	1	Identify the report as a systematic review.	Yes
**BACKGROUND**			
Objectives	2	Provide an explicit statement of the main objective(s) or question(s) the review addresses.	Yes
**METHODS**			
Eligibility criteria	3	Specify the inclusion and exclusion criteria for the review.	Yes
Information sources	4	Specify the information sources (e.g. databases, registers) used to identify studies and the date when each was last searched.	Yes
Risk of bias	5	Specify the methods used to assess risk of bias in the included studies.	Yes
Synthesis of results	6	Specify the methods used to present and synthesise results.	Yes
**RESULTS**			
Included studies	7	Give the total number of included studies and participants and summarise relevant characteristics of studies.	Yes
Synthesis of results	8	Present results for main outcomes, preferably indicating the number of included studies and participants for each. If meta-analysis was done, report the summary estimate and confidence/credible interval. If comparing groups, indicate the direction of the effect (i.e. which group is favoured).	Yes
**DISCUSSION**			
Limitations of evidence	9	Provide a brief summary of the limitations of the evidence included in the review (e.g. study risk of bias, inconsistency and imprecision).	Yes
Interpretation	10	Provide a general interpretation of the results and important implications.	Yes
**OTHER**			
Funding	11	Specify the primary source of funding for the review.	NA
Registration	12	Provide the register name and registration number.	Yes

Supplementary Table S2. PRISMA 2020 checklist

Section and Topic	Item #	Checklist item	Location where item is reported
**TITLE**			
Title	1	Identify the report as a systematic review.	Page 1
**ABSTRACT**			
Abstract	2	See the PRISMA 2020 for Abstracts checklist.	Page 2
**INTRODUCTION**			
Rationale	3	Describe the rationale for the review in the context of existing knowledge.	Page 5
Objectives	4	Provide an explicit statement of the objective(s) or question(s) the review addresses.	Page 5-6
**METHODS**			
Eligibility criteria	5	Specify the inclusion and exclusion criteria for the review and how studies were grouped for the syntheses.	Page 6-7
Information sources	6	Specify all databases, registers, websites, organisations, reference lists and other sources searched or consulted to identify studies. Specify the date when each source was last searched or consulted.	Page 6
Search strategy	7	Present the full search strategies for all databases, registers and websites, including any filters and limits used.	Page 6
Selection process	8	Specify the methods used to decide whether a study met the inclusion criteria of the review, including how many reviewers screened each record and each report retrieved, whether they worked independently, and if applicable, details of automation tools used in the process.	Page 7
Data collection process	9	Specify the methods used to collect data from reports, including how many reviewers collected data from each report, whether they worked independently, any processes for obtaining or confirming data from study investigators, and if applicable, details of automation tools used in the process.	Page 7
Data items	10a	List and define all outcomes for which data were sought. Specify whether all results that were compatible with each outcome domain in each study were sought (e.g. for all measures, time points, analyses), and if not, the methods used to decide which results to collect.	Page 7
	10b	List and define all other variables for which data were sought (e.g. participant and intervention characteristics, funding sources). Describe any assumptions made about any missing or unclear information.	Page 7
Study risk of bias assessment	11	Specify the methods used to assess risk of bias in the included studies, including details of the tool(s) used, how many reviewers assessed each study and whether they worked independently, and if applicable, details of automation tools used in the process.	Page 8
Effect measures	12	Specify for each outcome the effect measure(s) (e.g. risk ratio, mean difference) used in the synthesis or presentation of results.	Page 7-8
Synthesis methods	13a	Describe the processes used to decide which studies were eligible for each synthesis (e.g. tabulating the study intervention characteristics and comparing against the planned groups for each synthesis (item #5)).	Page 7-8

	13b	Describe any methods required to prepare the data for presentation or synthesis, such as handling of missing summary statistics, or data conversions.	Page 7-8
	13c	Describe any methods used to tabulate or visually display results of individual studies and syntheses.	Page 7-8
	13d	Describe any methods used to synthesize results and provide a rationale for the choice(s). If meta-analysis was performed, describe the model(s), method(s) to identify the presence and extent of statistical heterogeneity, and software package(s) used.	Page 7-8
	13e	Describe any methods used to explore possible causes of heterogeneity among study results (e.g. subgroup analysis, meta-regression).	Page 8
	13f	Describe any sensitivity analyses conducted to assess robustness of the synthesized results.	NA
Reporting bias assessment	14	Describe any methods used to assess risk of bias due to missing results in a synthesis (arising from reporting biases).	Page 8
Certainty assessment	15	Describe any methods used to assess certainty (or confidence) in the body of evidence for an outcome.	Page 8 (where applicable)
**RESULTS**			
Study selection	16a	Describe the results of the search and selection process, from the number of records identified in the search to the number of studies included in the review, ideally using a flow diagram.	Page 9
	16b	Cite studies that might appear to meet the inclusion criteria, but which were excluded, and explain why they were excluded.	Page 9
Study characteristics	17	Cite each included study and present its characteristics.	Page 11
Risk of bias in studies	18	Present assessments of risk of bias for each included study.	Page 9 and Supplementary
Results of individual studies	19	For all outcomes, present, for each study: (a) summary statistics for each group (where appropriate) and (b) an effect estimate and its precision (e.g. confidence/credible interval), ideally using structured tables or plots.	Page 11-12 and Supp.
Results of syntheses	20a	For each synthesis, briefly summarise the characteristics and risk of bias among contributing studies.	Page 9 + Supp.
	20b	Present results of all statistical syntheses conducted. If meta-analysis was done, present for each the summary estimate and its precision (e.g. confidence/credible interval) and measures of statistical heterogeneity. If comparing groups, describe the direction of the effect.	Page 11
	20c	Present results of all investigations of possible causes of heterogeneity among study results.	Page 11-12
	20d	Present results of all sensitivity analyses conducted to assess the robustness of the synthesized results.	Page 11
Reporting biases	21	Present assessments of risk of bias due to missing results (arising from reporting biases) for each synthesis assessed.	Page 9 and 11
Certainty of evidence	22	Present assessments of certainty (or confidence) in the body of evidence for each outcome assessed.	Page 11
**DISCUSSION**			
Discussion	23a	Provide a general interpretation of the results in the context of other evidence.	Page 16
	23b	Discuss any limitations of the evidence included in the review.	Page 16-17

	23c	Discuss any limitations of the review processes used.	Page 16
	23d	Discuss implications of the results for practice, policy, and future research.	Page 16-17
**OTHER INFORMATION**			
Registration and protocol	24a	Provide registration information for the review, including register name and registration number, or state that the review was not registered.	Page 7
	24b	Indicate where the review protocol can be accessed, or state that a protocol was not prepared.	Page 7
	24c	Describe and explain any amendments to information provided at registration or in the protocol.	Page 7-8
Support	25	Describe sources of financial or non-financial support for the review, and the role of the funders or sponsors in the review.	Page 18
Competing interests	26	Declare any competing interests of review authors.	Page 18
Availability of data, code and other materials	27	Report which of the following are publicly available and where they can be found: template data collection forms; data extracted from included studies; data used for all analyses; analytic code; any other materials used in the review.	Page 18

Supplementary Table S3. Results of quality assessment and risk of bias using JBI tools for critical appraisal of Case Reports. This table only includes reports of single patients or single entries in public databases.

ID	Record main info	Q1	Q2	Q3	Q4	Q5	Q6	Q7	Q8	Score	Quality	Risk of bias	Consensus	Notes
[5]	Al-Hamed 2020	Yes	Unclear	Yes	Yes	Yes	NA	Yes	Yes	6 / 8	Moderate	Low	Majority	Disagreement on Q2
[3]	Sakakibara 2022	Yes	No	Unclear	No	Yes	Yes	Yes	Yes	5 / 8	Moderate	Moderate	Unanimity	From a large cohort
[7]	Nigro 2023	Yes	Unclear	Unclear	Yes	Yes	NA	Yes	Yes	5 / 8	Moderate	Moderate	Majority	Disagreement on Q3
[8]	Findeisen 2025	Yes	No	Yes	Yes	Yes	Yes	Yes	Yes	7 / 8	High	Low	Majority	Disagreement on Q7
[16]	SCV007606611.1	No	No	Yes	Yes	Yes	Yes	Unclear	NA	4 / 8	Low	Moderate	Unanimity	Retrieve more info
[16]	SCV006333410.1	No	No	Yes	Yes	Yes	Yes	Unclear	NA	4 / 8	Low	Moderate	Unanimity	Retrieve more info
[16]	SCV002318839.1	No	No	Yes	Yes	Yes	Yes	Unclear	NA	4 / 8	Low	Moderate	Unanimity	Retrieve more info
[19]	Dzedzik 2026	Yes	Yes	Unclear	No	Yes	Yes	Unclear	Yes	5 / 8	Moderate	Moderate	Majority	Disagreement on Q3
[14]	Shamseldin 2020	Yes	Yes	No	Yes	Yes	Yes	Unclear	Unclear	5 / 8	Moderate	Moderate	Unanimity	VUS / LB
[20]	Aghjeh 2026	Yes	No	Unclear	Yes	No	No	No	Unclear	2 / 8	Low	High	Unanimity	VUS

Score: sum of all the "Yes" answers. **Quality:** Low (0-4), Moderate (5-6), High (7-8). **Risk of bias:** Low (maximum 1 answer with "Unclear" or "No"); Moderate (2-3 answers with "Unclear" or "No"); High (more than 3 answers with "Unclear" or "no").

Q1: Were patient's demographic characteristics clearly described?

Q2: Was the patient's history clearly described and presented as a timeline (*or at least with age of onset for manifestations*)?

Q3: Was the current clinical condition of the patient clearly defined and investigated for differential diagnosis? *

Q4: Were diagnostic genetic tests and the results clearly described, with official ACMG classification? *

Q5: Was specific assessment for extrarenal features performed? *

Q6: Was the extrarenal feature clearly defined or described? *

Q7: Were comorbidities or unanticipated events that could explain extrarenal features excluded? *

Q8: Does the case report provide takeaway lessons?

The "*" indicates questions that were modified from the original JBI templates (e.g. no pharmacological intervention involved).

Supplementary Table S4. Results of quality assessment and risk of bias using JBI tools for critical appraisal of Case Series. This table only includes reports of 2 or more patients with a MAPKBP1-related disease.

Ref	Author and Year	Q1	Q2	Q3	Q4	Q5	Q6	Q7	Q8	Q9	Q10	Score	Quality	Risk of bias	Consensus	Notes
[4]	Macia 2017	Y	Y	Y	Y	Y	Y	Y	U	Y	NA	8 / 10	Moderate	Low	Majority	Disagreement on Q8
[6]	Schonauer 2020	Y	Y	Y	Y	Y	Y	Y	U	Y	NA	8 /10	Moderate	Low	Unanimity	Ehlers-Danlos?

Y = Yes. N = No. U = Unclear. NA = Not Applicable.

Score: sum of all the "Yes" answers. **Quality:** Low (0-5), Moderate (6-8), High (9-10). **Risk of bias:** Low (maximum 2 answer with "Unclear" or "No"); Moderate (3-4 answers with "Unclear" or "No"); High (more than 4 answers with "Unclear" or "no").

Q1: Were there clear criteria for inclusion in the case series or was the series homogenous? *

Q2: Was the condition measured/diagnosed in a standard, reliable way for all participants included in the case series?

Q3: Were valid methods used for genetic testing and for the exclusion of differential diagnosis? *

Q4: Did the case series have consecutive and complete inclusion of participants? *

Q5: Was specific assessment for extrarenal features performed? *

Q6: Was there clear reporting of the demographics of the participants in the study?

Q7: Was there clear reporting of clinical information of the participants?

Q8: Were comorbidities or unanticipated events that could explain extrarenal features excluded? *

Q9: Was there clear reporting of the presenting site(s)/clinic(s) demographic information (e.g. same family; consanguinity)?

Q10: Was statistical analysis appropriate?

The "*" indicates questions that were modified from the original JBI templates (e.g. no pharmacological intervention involved).

For details, see: 1) Munn Z, Barker T, Moola S, Tufanaru C, Stern C, McArthur A, Stephenson M, Aromataris E. Methodological quality of case series studies, JBI Evidence Synthesis, doi: 10.11124/JBISRIR-D-19-00099. **2)** Moola S, Munn Z, Tufanaru C, Aromataris E, Sears K, Sfetcu R, Currie M, Qureshi R, Mattis P, Lisy K, Mu P-F. Chapter 7: Systematic reviews of etiology and risk. In: Aromataris E, Munn Z (Editors). JBI Manual for Evidence Synthesis. JBI, 2020.
